# Cross-Regulation between Autophagy and Apoptosis Induced by Vitamin E and Lactobacillus Plantarum through Beclin-1 Network

**DOI:** 10.3390/ijms232315305

**Published:** 2022-12-04

**Authors:** Ahlam M. Alhusaini, Sara A. Alhumaidan, Ghaida M. Alharbi, Eman A. Alzahrani, Wedad S. Sarawi, Hatun A. Alomar, Abeer M. Alanazi, Dareen S. Mattar, Iman H. Hasan

**Affiliations:** 1 Department of Pharmacology and Toxicology, College of Pharmacy, King Saud University, P.O. Box 22452, Riyadh 11495, Saudi Arabia; 2 College of Pharmacy, King Saud University, P.O. Box 22452, Riyadh 11495, Saudi Arabia; 3 Department of Physiology, College of Medicine, Umm Al Qura University, P.O. Box 715, Makkah 21955, Saudi Arabia

**Keywords:** autophagy, apoptosis, vitamin E, Lactobacillus-plantarum, Beclin-1

## Abstract

Autophagy and apoptosis are two important regulatory mechanisms for how the body can respond to diseases. This study was designed to investigate the protective actions of vitamin E (Vit-E) and lactobacillus plantarum (Lac-B) against mercuric chloride (HgCl_2_)-induced kidney injury. Thirty albino rats were divided into five groups: group 1 served as the normal group; rats in group 2 received high doses of HgCl_2_; rats in groups 3, 4 and 5 were given Vit-E, Lac-B and the combination of Vit-E and Lac-B, respectively along with HgCl_2_ for two weeks. HgCl_2_ provoked renal injury, manifested by elevation in serum urea, urea nitrogen and creatinine. Kidney levels of oxidative stress and inflammation were markedly increased post HgCl_2_ administration. Moreover, HgCl_2_ significantly elevated the gene expression levels of VCAM-1 and cystatin *C*, while podocin was downregulated. Additionally, it markedly decreased the protein expression of Beclin-1 and Bcl-2. Histopathological examination revealed massive degeneration with congested blood vessels following HgCl_2_ administration. Treatment with Vit-E or/and Lac-B restored the normal levels of the previously mentioned parameters, as well as improved the morphology of kidney tissues. Both Vit-E and Lac-B provided a protective effect against HgCl_2_-induced kidney damage by regulating autophagy and apoptosis.

## 1. Introduction

Autophagy is a crucial part of cell function and regulation through which it removes misfolded proteins, clears dysfunctional organelles and eliminates pathogens and harmful cells [1]. During autophagy, intracellular organelles and parts of the cytosol are initially isolated from the cytoplasm in an autophagic vacuole, which then merges with lysosomes to create an auto-phagolysosome, which is subsequently digested by lysosomal enzymes [1]. Apoptosis is a process of programmed cell death used to maintain homeostasis in the body; for instance, to eliminate cells that are no longer needed, programmed destruction of cells during embryogenesis and pathologic states such as the elimination of cells that are injured beyond repair. The mechanism of apoptosis is extremely complex and involves two main pathways, which ultimately result in the activation of caspases, a family of cysteine protease that have proteolytic activity [2]. Both autophagy and apoptosis are two cellular degradation mechanisms that are critical for maintaining organismal homeostasis. Many important molecules, including members of the Bcl-2 family, Beclin-1 and apoptosis-related proteins such as caspases, mediate the cross-regulation between autophagy and apoptosis [3].

The dysregulation of autophagy and apoptosis is implicated in many pathological states, including many diseases, aging, cancer and heavy metals toxicities [4,5]. It is well known that reactive oxygen species (ROS) production is mediated by phagocytic cells as their role in cell defense mechanisms. ROS can initiate cell death mainly by DNA damage, also they have a role in stimulating autophagic cell death, helping the cell to eliminate the oxidizing components [6]. Excessive production of ROS can provoke either cell survival or apoptosis mechanisms according to the severity and duration of exposure.

Nowadays, using antioxidants has become a useful therapeutic approach against different pathological disorders. Vitamin E (Vit-E) is a fat-soluble vitamin known for its antioxidant, anti-inflammatory and cytoprotective activities. Vit-E also plays an important role in the stability of membrane permeability and fluidity [7]. Metal ion-induced renal damage is caused by significant lipid peroxidation, which can be reduced with Vit-E pretreatment [7]. Lactobacillus plantarum (Lac-B) is a group of gram-positive, rod-shaped bacteria that can live in both aerobic and anaerobic conditions. Lac-B is known for producing lactic acid as a byproduct of glucose metabolism. Their protective effects could be attributed to their antioxidant property, more specifically their ability to decrease the risk of ROS accumulation [8] and subsequent cell death mechanisms. Vit-E has a critical role in suppressing tumor cells through controlling proliferation, differentiation and apoptosis [9]. Moreover, in a recently published review, Rakowski and colleagues have discussed the autophagic regulatory actions of Vit-E supplementation in many diseases including diabetic nephropathy, depression and hepatocellular carcinoma [10]. Likewise, Lac-B showed an inhibitory effect mitochondrial-mediated apoptosis pathway in hepatocytes [11].

The interaction between autophagy and apoptosis in cell death is very complex and unpredictable. Both mechanisms are involved in heavy metal-induced cell death; however, they can either antagonize or synergize each other based on the tissues and the toxins used. This raised our curiosity to investigate autophagy and apoptosis interaction in Hg toxicity and how it can be modulated with the use of antioxidants like Lac-B and Vit-E. To the best of our knowledge, there is no previous study addressing the crosslink of apoptosis and autophagy after Hg toxicity and with antioxidant interventions. Thus, we hypothesized that Vit-E and Lac-B have nephroprotective activity against HgCl_2_-induced kidney damage via maintaining the hemostasis between autophagy and apoptosis through modulation of Beclin-1 and Bcl-2 cross-regulation; hence, improving the autophagy/apoptosis flux could become a potential therapeutic target.

## 2. Results

### 2.1. Vit-E and Lac-B Restored Renal Function after HgCl_2_ Induced Kidney Injury

**Figure 1:**
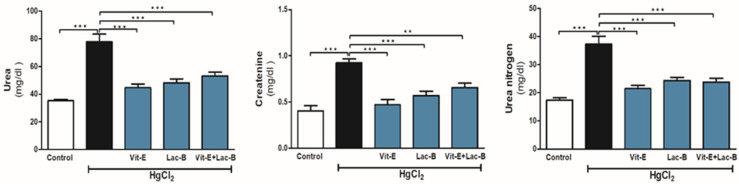
Vit-E and Lac-B prevented HgCl_2_-induced renal injury in rats. Treatments with Vit-E and Lac-B either alone or in combination ameliorated serum urea, urea nitrogen and creatinine levels. Data are expressed as mean ± SEM, (*n* = 6). ** *p* < 0.01 and *** *p* < 0.001.

Serum levels of urea, urea nitrogen and creatinine were assessed after HgCl_2_ toxicity with and without treatments, as shown in Figure 1. As expected, HgCl_2_ administration showed a significant elevation in serum urea, creatinine and urea nitrogen levels (*p* ≤ 0.001). These parameters were significantly lowered to their average values by using Vit-E and Lac-B either alone or in combination (*p* ≤ 0.01 and *p* ≤ 0.001, relative to HgCl_2_ intoxicated group).

### 2.2. Vit-E and Lac-B Mitigated Oxidative Stress after HgCl_2_ Induced Kidney Injury

**Figure 2:**
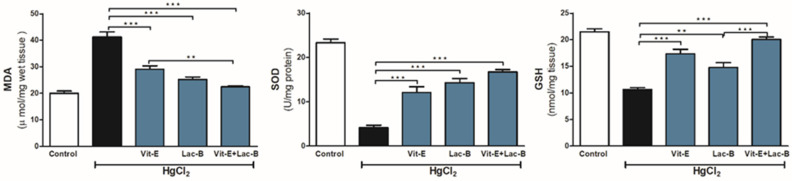
Vit-E and Lac-B attenuated HgCl_2_-induced renal injury in rats. Treatments with Vit-E and Lac-B either alone or in combination decreased renal MDA and increased GSH and SOD. Data are expressed as mean ± SEM, (*n* = 6). ** *p* < 0.01 and *** *p* < 0.001.

The antioxidant effects of Vit-E and Lac-B were evaluated against the renal oxidative stress induced by HgCl_2_ (Figure 2). The metal intoxicated group showed an increase in oxidative stress markers in the renal tissues, namely MDA (*p* ≤ 0.001), and a decrease in SOD (*p* ≤ 0.001) and GSH (*p* ≤ 0.001) levels in comparison to controls. Furthermore, single, or simultaneous use of Vit-E and Lac-B possessed a remarkable antioxidant effect, reducing MDA (*p* ≤ 0.001) and increasing both SOD (*p* ≤ 0.001) and GSH (*p* ≤ 0.001) levels relative to the intoxicated group. Notably, more protective effect was obtained after using Vit-E + Lac-B treatment and thus confirmed the benefits of combined treatment.

### 2.3. Vit-E and Lac-B Attenuated the HgCl_2_ Nephrotoxicity via Increasing Beclin-1 and Bcl-2 Protein Expression

**Figure 3:**
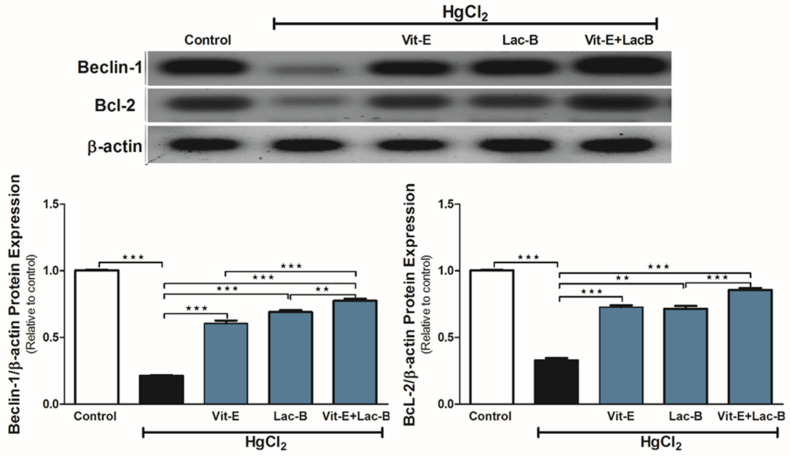
Vit-E and Lac-B upregulated renal Beclin-1 and Bcl-2 expression in HgCl_2_-intoxicated rats. Representative blots show changes in the expression of Bcl-2 and Beclin-1 in all treated groups. Data were expressed as mean ± SEM, (*n* = 6). ** *p* < 0.01 and *** *p* < 0.001.

The heavy metal administration altered the renal expression of Beclin-1 and Bcl-2 proteins (Figure 3), in which both proteins were significantly downregulated (*p* ≤ 0.001) in comparison to the control rats. The use of Vit-E and Lac-B on their own or in combination significantly ameliorated such protein alterations and relatively restored the expression level of control group, especially with the treatment combination.

### 2.4. Vit-E and Lac-B Diminished the HgCl_2_ Nephrotoxicity via Modulating Cystatin C, VCAM-1 and Podocin Genes

**Figure 4:**
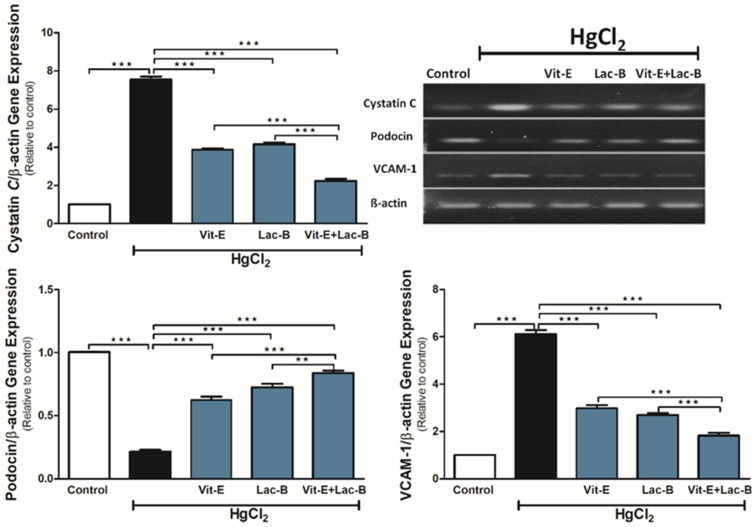
Vit-E and Lac-B suppressed renal *cystatin C*, VCAM-1 and increased podocin mRNA expression in HgCl_2_-intoxicated rats. Representative blots are showing variations in the expression of cystatin *C,* podocin, VCAM-1 and β-actin in all treated groups. Data were expressed as mean ± SEM, (*n* = 6). ** *p* < 0.01 and *** *p* < 0.001.

The exposure to HgCl_2_ caused a significant upregulation in *cystatin C* (*p* ≤ 0.001) and *VCAM-1* (*p* ≤ 0.001) and downregulation in podocin (*p* ≤ 0.001) gene expression (Figure 4) relative to the control group. Vit-E or Lac-B significantly ameliorated HgCl_2_ effects on these genes (*p* ≤ 0.001) by restoring their regular expression levels. Concurrent use of Vit-E and Lac-B showed further additive effects on these genes compared to single treatment (*p* ≤ 0.01, *p* ≤ 0.001).

### 2.5. Vit-E and Lac-B Attenuated Renal Tissue Inflammation after HgCl_2_ Exposure

**Figure 5:**
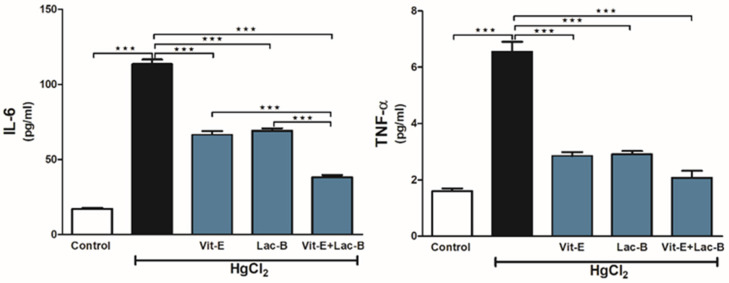
Vit-E and Lac-B attenuated HgCl_2_-induced renal inflammation in rats. Treatment with Vit-E and Lac-B either alone or in combination decreased renal inflammatory biomarkers (IL-6 and TNF-α). Data are expressed as mean ± SEM, (*n* = 6). *** *p* < 0.001.

HgCl_2_ provoked renal tissue inflammatory response confirmed by the significant increase in renal IL-6 (*p* ≤ 0.001) and TNF-α (*p* ≤ 0.001), as shown in Figure 5. Treatment with Vit-E and/or Lac-B significantly reduced the levels of these markers in the kidneys of HgCl_2_-intoxicated rats.

### 2.6. Vit-E and Lac-B Inhibited Renal Apoptotic Cell Damage in Response to HgCl_2_

**Figure 6:**
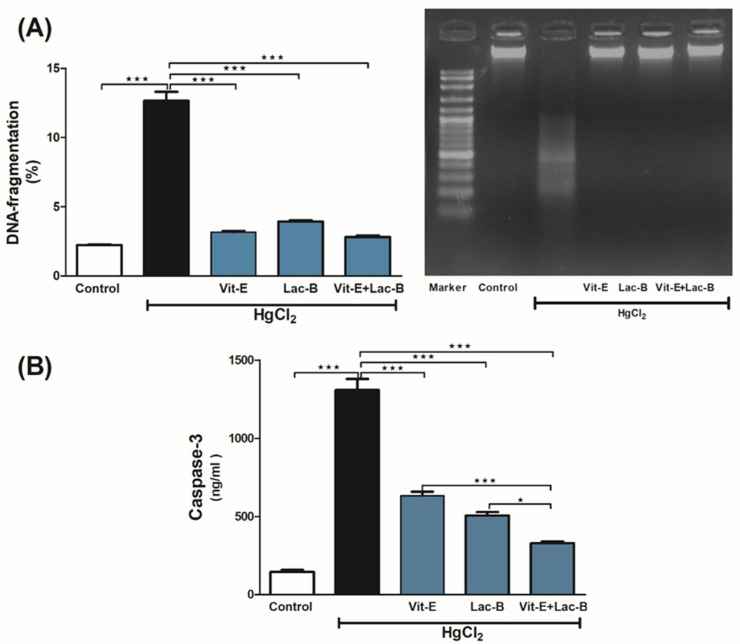
Vit-E and Lac-B suppressed renal apoptotic damage in HgCl_2_-intoxicated rats. (**A**) Representative blot is showing variations in the DNA fragmentation in all treated groups, (**B**) changes in renal tissue expression of Caspase 3 in all treatment groups. Data are expressed as mean ± SEM, (*n* = 6). *** *p* < 0.001, * *p* < 0.05.

Genomic DNA integrity and caspase-3 expression were used to assess the protective effects of Vit-E and Lac-B. In agarose gel, the intoxicated rats showed a smeared band which reflected DNA fragmentation; however, Vit-E and Lac-B were effective against HgCl_2_ -induced cell damage and kept the DNA intact (Figure 6A). Besides, Vit-E and Lac-B possessed anti-apoptotic action by significantly reducing Caspase-3 expression (*p* ≤ 0.001) that had been elevated in response to HgCl_2_ (Figure 6B).

### 2.7. Vit-E and Lac-B Ameliorated HgCl_2_ Induced Histopathological Changes in Renal Tissue

**Figure 7:**
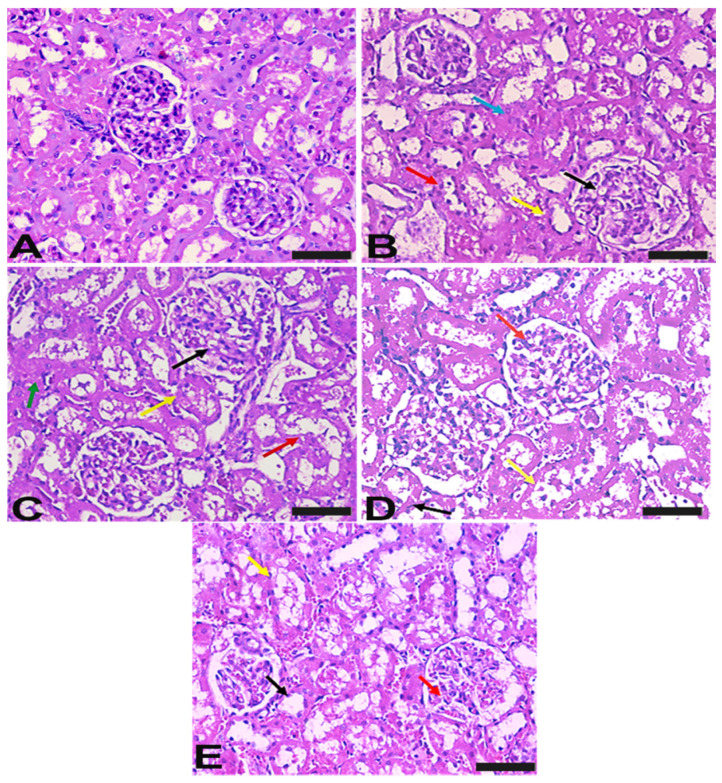
Photomicrograph of H&E-stained sections of rat kidney. (**A**) section from normal control rat shows normal histological appearance of renal corpuscle with normal glomerulus (black arrow). (**B**) section of kidney from rat received HgCl_2_ showed few of the glomeruli corpuscles are obliterated and destructed (hyperplasia of epithelial cells lining the partial layer of Bowman’s capsule) (black arrow). Proximal convoluted tubules show destructed epithelial lining (yellow arrow), destructed epithelial lining of distal convoluted tubules (red arrow), (**C**) section of kidney from HgCl_2_ administrated rat treated by Vit-E showed few of the glomeruli corpuscles are obliterated and destructed (hyperplasia of epithelial cells lining the partial layer of Bowman’s capsule) (black arrow). Proximal convoluted tubules show the destructed epithelial lining (yellow arrow), destructed epithelial lining of distal convoluted tubules (red arrow). (**D**) section from HgCl_2_ administrated rat received Lac showed renal cortex showing renal corpuscle with almost normal glomerulus (red arrow), mildly dilated proximal convoluted (black arrow) and mildly dilated distal convoluted (yellow arrow) tubules. (**E**) section of kidney from HgCl_2_ administrated rat treated by (Vit-E+ Lac) showed renal cortex showing renal corpuscle with normal glomerulus (red arrow), normal pattern of proximal convoluted (black arrow) and distal convoluted (yellow arrow) tubules (Scale bar; 400 µm).

The histological staining was used to evaluate the pathological changes that occurred after HgCl_2_ exposure and how they responded to treatments (Figure 7). In contrast to the normal renal architecture in controls, toxicity induced degenerated glomeruli corpuscles, obliterated hyperplasia and destructed tubules. Treatment with Vit-E improved the tissue appearance via revealing fewer glomeruli and tubular damage. In addition, Lac-B revealed almost normal glomerulus with mild tubule dilatation. Combined treatments showed normal glomerulus and tubules and reversed the pathological changes associated with HgCl_2_ administration.

## 3. Discussion

The findings of this study provide new evidence of the importance of the cross-regulation between autophagy and apoptosis in the attenuation of HgCl_2_-induced renal toxicity by using Vit-E or/and Lac-B. Autophagy is a programmed and self-catabolic cellular mechanism that removes impaired proteins and organelles [12]. It can be induced in response to a wide range of stressors to protect the cells from protein aggregation and lipid oxidation. The dysregulation of autophagy is associated with many diseases, aging and cancer [5]. Apoptosis is a process in which the cell shrinks, the chromatin condenses and at the end of the process, the cell divides into pieces called ‘apoptotic bodies’ [6]. Thus, the primary target of autophagy is the cytoplasm, while the main target of apoptosis is the nucleus [6]. No matter what kind of cell death, caused by either autophagy or apoptosis, both processes are regulated by molecular mechanisms.

In the current study, the daily administration of high dose of HgCl_2_ for two weeks markedly decreased the protein expression of Beclin-1 and Bcl-2, increased caspase-3 expression and increased DNA fragmentation. Beclin-1 is a specific protein for regulating autophagy, while Bcl-2 is a protein responsible for cell persistence and acts as antiapoptotic protein. It is well-known that Beclin-1protein can interact with a Bcl-2 protein and the latter protein can modulate either apoptosis or autophagy via its interaction with Beclin-1 [13]. Thus, in human pathogenic cells, the levels of Beclin-1 and Bcl-2 proteins indicate the presence of a dysfunctional system of autophagy/apoptosis [14]. Therefore, the cross-regulation between autophagy and apoptosis has become an important topic that is mostly explained by the interaction between Bcl-2 and Beclin-1. In normal conditions, Bcl-2 reduces autophagy through interacting with Beclin-1/BH-3 domain [12]. Moreover, the downregulation of Beclin-1 is thought to inhibit autophagy and prevent damaged mitochondria’s turnover, leading to ROS production and stress [12]. It was shown by Araragi and his colleagues that HgCl_2_ could induce apoptosis through activation of caspase-3 (pro-apoptotic signal) and inducing of DNA fragmentation [4]. Interestingly, cleavage of Beclin-1 mediated by caspase-3 is another mechanism that promotes the crosstalk between autophagy and apoptosis [6]. Conversely, it has been reported that hepatic cells exposed to a small concentration of HgCl_2_ activated autophagic cell death to serve a protective mechanism [15]. Many studies showed that autophagy is induced in different cells exposed to low concentrations of heavy metals [16,17,18].

The administration of HgCl_2_ caused a significant increase in oxidative stress (increasing renal MDA level and decreasing GSH level and SOD activity) and inflammatory biomarkers (increasing of IL-6 and TNF-α levels). Oxidative stress is a phenomenon that is caused by the accumulation of ROS under the stimulation of various physicochemical factors [19]. Accumulating evidence showed the interconnection between the oxidative stress and the progression of many diseases and the production of ROS could be increased during the early stage of inflammation as a body defense mechanism [20]. In cell biology, it is a well-known that ROS have both positive and negative effects on the cell. A very low level of ROS can function as the second runner in some signaling pathways, as there is a dynamic relation between the production of ROS and the endogenous antioxidant capacity [21]. Nevertheless, they may cause oxidative stress in most body systems when they are produced at excessive concentrations [6]. The production of ROS can initiate cell death mainly by DNA damage, also they have a role in stimulating autophagic cell death helping the cell to eliminate the oxidizing components [6]. Recent studies revealed that the large dose of HgCl_2_ caused elevation of oxidative stress and inflammatory markers in different body organs [22,23]. Moreover, Fouda et al. showed a significant increase of renal oxidative stress and apoptosis post subcutaneous administration of HgCl_2_ [24].

Our study revealed a significant elevation of VCAM-1 and cystatin *C* mRNA expression levels following HgCl_2_ administration. The expression of VCAM-1 is shown to be activated by ROS and pro-inflammatory cytokines such as TNF-α [25]. Recent study suggested that VCAM-1 is an appropriate biomarker for kidney injury [26]. TNF-α belongs to TNF superfamily, which is mainly produced by macrophages and lymphocytes to regulate immunologic and pro-inflammatory activities [27]. It has been shown that the activation of autophagy is critical for VCAM-1 expression [28]. This study showed a marked downregulation of podocin expression post HgCl_2_ administration. Podocin is a protein localized in the podocyte membrane and it is functioning as a regulator of the glomerular permeability [29]. The absence of this protein indicates a severe and early onset of nephrotic syndrome [29]. It has been shown that acute administration of HgCl_2_ to mice caused down-regulation of podocin, leading to acute kidney injury [30].

The elevated levels of oxidative stress, apoptotic and proinflammatory biomarkers and DNA fragmentation were reduced upon using Vit-E or/and Lac-B. These antioxidants ameliorated the abnormal expression of VCAM-1, cystatin C and podocin gene expressions. Additionally, treatment with Vit-E or/and Lac-B significantly restored the protein expression of Beclin-1 and Bcl-2 to their normal levels. Recent study revealed the protective effect of Lac-B against Zearalenone-induced kidney genotoxicity through modulation of MDA, GSH peroxidase, IL-6, IL-10, TNF-α, caspase-3 and Bcl-2 levels, as well as reduction of DNA fragmentation [31]. A clinical study showed an improvement in the renal function in patients with chronic kidney disease after Lac-B administration through decreasing of serum levels of TNF-α and IL-6 [32].

Nowadays, using antioxidants is a useful therapy approach against different pathological disorders. Thus, the findings of this study suggested that Vit-E and Lac-B are potent inducers of the autophagy/apoptosis-regulatory mechanisms through diverse molecular pathways including Beclin-1, Bcl-2 and caspase-3 and these regulatory mechanisms can maintain renal cellular homeostasis.

## 4. Materials and Methods

### 4.1. Chemicals

Vit-E and HgCl_2_ were purchased from Sigma Chemical Co. (Sigma-Aldrich, St. Louis, MO, USA). Lac-B was obtained from a local pharmacy. The primary antibodies of vascular cellular adhesion molecule-1 (VCAM-1), *cystatin C*, podocin, Beclin-1 and B-cell lymphoma 2 (Bcl-2) were obtained from Santa Cruz (Santa Cruz Biotechnology, Dallas, TX, USA).

### 4.2. Animals

Thirty male Wistar albino rats weighing 150–200 g were obtained from the Bio-Resource Unit, College of Pharmacy, King Saud University. Animals were fed with standard rat pellet chow with free access to water ad libitum. The study was conducted in accordance with the Basic & Clinical Pharmacology & Toxicology policy for experimental and clinical studies [33]. The Experimental protocol was approved by the Research Ethics Committee, King Saud University (KSU-SE-19-38).

### 4.3. Experimental Design

Rats were randomly divided into five groups, six rats/group; they were treated as follows: the first group was served as the normal control group; in the second group, rats were intoxicated subcutaneously with 5 mg/kg HgCl_2_ once daily for two weeks [34]; the rats in the third group were treated with Vit-E at a dose of 100 mg/kg/day, orally [35]; the fourth group was orally treated with 6 × 10^10^ CFU of Lac-B at a dose of 1.8701/kg in 1 mL normal saline [36]; and the fifth group was treated with the combination of Vit-E and Lac-B. All treatments were given daily along with HgCl_2_ for two weeks.

After completing of all treatments, rats were subjected to a gradual concentration of CO_2_, then sacrificed by decapitation. Blood samples were collected, and sera were separated by centrifugation at 3000 rpm for 20 min. The kidneys were also collected; parts of the kidneys were homogenized in phosphate buffer to yield 20% homogenates. Then the homogenates were centrifuged for 20 min at 3000 rpm at 4 °C and the supernatants were kept at −80 °C. Other parts of the kidneys were rapidly frozen under liquid nitrogen and stored at −80 °C for western blotting. Parts of kidney tissues from each group were kept in 10% formalin for histopathological examination.

### 4.4. Biochemical Analyses

#### 4.4.1. Serum Creatinine, Urea and Urea Nitrogen

According to the manufacturer’s instructions, creatinine, urea and urea nitrogen levels in the serum were evaluated using colorimetric assay from Randox Company (London, UK).

#### 4.4.2. Oxidative Stress and Antioxidant Defenses

MDA was determined in the kidney homogenate as previously described [37]. GSH level and SOD activity were assayed according to the methods of Ellman [38] and Marklund and Marklund [39], respectively.

#### 4.4.3. Inflammatory and Apoptotic Markers

The expression levels of pro-inflammatory markers (tumor necrosis factor alpha (TNF-α) and interleukin 6 (IL-6)) and the apoptotic marker (cysteine–aspartic acid protease 3 (caspase-3)) were measured in kidney homogenate using highly sensitive ELISA kits obtained from Immuno-Biological Laboratories (Takasaki-Shi, Gunma, Japan).

#### 4.4.4. DNA Fragmentation

Agarose electrophoresis and the colorimetric methods [40] were used to assess DNA fragmentation. The results were presented as a fold change of the control.

#### 4.4.5. Protein Expression

Western blots were performed to determine the protein expressions of Beclin-1 and Bcl-2. The protein bands were visualized by ImageQuant LAS-4000 (GE Healthcare, USA) then quantified using ImageJ.

#### 4.4.6. Gene Expression

The effect of HgCl_2_ and treatment agents on the expressions of renal VCAM-1, *cystatin C* and podocin were determined by RT-PCR as previously described [41]. Amplification of cDNA was carried out using SYBR green master mix (Thermo Fisher Scientific, Waltham, MA, USA) in the presence of target primers (Table 1). The signal amplification was then detected and quantified using ABI 7500 real-time PCR System (Applied Biosystems, USA). The obtained data were analyzed using the 2^−ΔΔCt^ method [41] and normalized to β-actin.

#### 4.4.7. Histological Examination

The kidney samples, fixed in 10% formaldehyde, were dehydrated, embedded in paraffin and cut into 5-µm thick-sections. The prepared sections were processed for staining with hematoxylin and eosin (H&E) and were examined using a light microscope.

### 4.5. Statistical Analysis

The results were expressed as mean ± standard error of the mean (SEM). Statistical analysis and multiple comparisons were performed by one-way ANOVA followed by Tukey’s post-hoc test using Graph Pad Prism 5. The result was considered significant if *p* value < 0.05.

## Figures and Tables

**Table 1:** Primer sequences.

Gene Name	Primer Code
*VCAM-1*	Forward: 5′- GAA TTC TCC CAA ATC GAC ATA TTC CC-3′ Reverse: 5′- CTC GAG TTA TTT CTC TTG AAC AGT TAA TT-3′
*podocin*	Forward: 5′- CCT GTG AGT GGC TTC TTG TCC TC-3′ Reverse: 5′- GGA GAC GCT TCA TAG TGG TTT GCA-3′
*cystatin C*	Forward: 5′- GCGTACCACAGCCGCGCCAT-3′ Reverse: 5′- TGGGGCTGGTCATGGAAAGGACAGT-3′
*β-actin*	Forward: 5′- GCA CCA CAC CTT CTA CAA TG-3′ Reverse: 5′- TGC TTG CTG ATC CAC ATC TG-3′

## Data Availability

The original contributions presented in the study are included in the article, further inquiries can be directed to the corresponding author.
